# Resveratrol regulates neuro-inflammation and induces adaptive immunity in Alzheimer’s disease

**DOI:** 10.1186/s12974-016-0779-0

**Published:** 2017-01-03

**Authors:** Charbel Moussa, Michaeline Hebron, Xu Huang, Jaeil Ahn, Robert A. Rissman, Paul S. Aisen, R. Scott Turner

**Affiliations:** 1 Department of Neurology, Laboratory for Dementia and Parkinsonism, Translational Neurotherapeutics Program, National Parkinson’s Foundation Center of Excellence, Georgetown University Medical Center, 4000 Reservoir Road, NW, Washington DC, 20057 USA; 2 Department of Neurology, Memory Disorders Program, Translational Neurotherapeutics Program, Georgetown University, Washington DC, USA; 3 Department of Biostatistics, Georgetown University Medical Center, 4000 Reservoir Road, NW, Washington DC, 20057 USA; 4 Alzheimer’s Therapeutic Research Institute (ATRI), University of Southern California, San Diego, CA USA; 5 Alzheimer’s Disease Cooperative Study (ADCS), Department of Neurosciences, University of California, La Jolla, San Diego, CA USA

**Keywords:** Resveratrol, Matrix metalloproteinase-(MMP)-9, Alzheimer, Interleukin-4, Macrophage-derived chemokine (MDC)

## Abstract

**Background:**

Treatment of mild-moderate Alzheimer’s disease (AD) subjects (*N* = 119) for 52 weeks with the SIRT1 activator resveratrol (up to 1 g by mouth twice daily) attenuates progressive declines in CSF Aβ40 levels and activities of daily living (ADL) scores.

**Methods:**

For this retrospective study, we examined banked CSF and plasma samples from a subset of AD subjects with CSF Aβ42 <600 ng/ml (biomarker-confirmed AD) at baseline (*N* = 19 resveratrol-treated and *N* = 19 placebo-treated). We utilized multiplex Xmap technology to measure markers of neurodegenerative disease and metalloproteinases (MMPs) in parallel in CSF and plasma samples.

**Results:**

Compared to the placebo-treated group, at 52 weeks, resveratrol markedly reduced CSF MMP9 and increased macrophage-derived chemokine (MDC), interleukin (IL)-4, and fibroblast growth factor (FGF)-2. Compared to baseline, resveratrol increased plasma MMP10 and decreased IL-12P40, IL12P70, and RANTES. In this subset analysis, resveratrol treatment attenuated declines in mini-mental status examination (MMSE) scores, change in ADL (ADCS-ADL) scores, and CSF Aβ42 levels during the 52-week trial, but did not alter tau levels.

**Conclusions:**

Collectively, these data suggest that resveratrol decreases CSF MMP9, modulates neuro-inflammation, and induces adaptive immunity. SIRT1 activation may be a viable target for treatment or prevention of neurodegenerative disorders.

**Trial registration:**

ClinicalTrials.gov NCT01504854

## Background

Increasing age is the primary risk factor for Alzheimer’s disease (AD), even in individuals with high genetic risk. The mild stressor caloric restriction (CR)—or consuming ~2/3 normal daily calories—postpones and prevents diseases of aging in animal models and perhaps also in man. In contrast, diabetes mellitus and caloric excess (obesity, particularly during midlife) accelerate the onset of AD, suggesting a link between glucose/energy metabolism and amyloid precursor protein/β-amyloid (Aβ) metabolism. While the mechanism of CR benefits remains unclear, activation of sirtuins, notably SIRT1, may be a critical molecular pathway. SIRT1 deacetylase activity is regulated by NAD+/NADH—coupling cellular energy balance to epigenetic transcriptional regulation. Resveratrol, a potent SIRT1 activator and pharmacologic mimic of CR, is a polyphenol found naturally in red grapes, peanuts, and many other plant species. Similar to CR, treatment of transgenic mouse models of AD with resveratrol decreases behavioral deficits and central nervous system (CNS) Aβ deposition with aging [1].

We hypothesized that molecular mechanisms of aging, specifically SIRT1, may be exploited as a target for development of AD therapeutics. Given the proven safety of resveratrol and promising preclinical data, we enrolled 119 subjects in a phase 2 randomized, double-blind, placebo-controlled trial of resveratrol in subjects with mild-moderate AD (with dosage stepped up to 2 g pure, synthetic resveratrol by mouth daily, for 12 months) [2]. High-dose oral resveratrol treatment is safe and well-tolerated—the only significant adverse effect is weight loss. Low nanomolar native resveratrol is detectable in cerebrospinal fluid (CSF), suggesting CNS penetration and a high-affinity molecular target (or targets). Compared to placebo, resveratrol stabilizes the progressive decline in CSF Aβ40 and plasma Aβ40 levels as dementia advances. In individuals with biomarker-confirmed AD (CSF Aβ42 <600 ng/ml) at baseline, resveratrol also stabilizes CSF Aβ42 levels [2]. Despite the phase 2 trial being underpowered to detect clinical benefits, resveratrol attenuated decline in the Alzheimer’s Disease Cooperative Study-Activity of Daily Living (ADCS-ADL) score during the 12-month study. Aging is also a major risk factor for cancer, and fewer cancers were found in the resveratrol-treated group (one versus seven cancers in six participants in the placebo group). Collectively, these data support the notion that targeting molecular mechanisms of aging may point to therapeutic strategies that postpone or prevent diseases of aging—in parallel. With proven safety and suggestions of efficacy in the phase 2 trial, the putative benefits of resveratrol and other sirtuin activator compounds (STACs) should be further examined in clinical studies.

Paradoxically, resveratrol treatment increased brain volume loss in AD subjects, compared to the placebo-treated group. Since CSF tau and phospho-tau levels are unaffected (suggesting no treatment effect on neuronal loss), we hypothesize that resveratrol has potent anti-inflammatory effects in AD brain—with decreased CNS edema as the etiology of greater brain volume loss. Similar effects are found with anti-amyloid immunotherapies for AD [3] and effective drugs for multiple sclerosis (MS) are also known to be associated with “pseudoatrophy” [4]. To test the putative anti-inflammatory effects of resveratrol in AD brain, we measured pro- and anti-inflammatory cytokines and chemokines, and metalloproteinases, in banked samples of CSF and plasma from a subset of individuals with biomarker-confirmed AD (CSF Aβ42 <600 ng/ml) in the phase 2 trial. Consistent with our hypothesis, we found significant anti-inflammatory effects of resveratrol in the CSF of treated AD subjects. Our data also suggest that resveratrol treatment preserved the integrity of the blood-brain barrier (BBB) in AD. Collectively, these exploratory findings lend support to the notion that targeting molecular pathways of aging may lead to novel therapies to postpone or prevent diseases of aging, including AD.

## Methods

### Patient demographics

With the Alzheimer’s Disease Cooperative Study, we recently completed a randomized, placebo-controlled, double-blind, multi-site, phase 2 trial of resveratrol in individuals with mild to moderate dementia due to AD [2]. The study drug was pure, synthetic resveratrol powder (encapsulated) versus matching placebo. Concomitant use of FDA-approved medications for AD (e.g., cholinesterase inhibitors) was allowed. The two randomized groups were similar at baseline with the exception that duration of diagnosis was longer in the placebo group. Participants (total *N* = 119) were randomized to placebo or resveratrol 500 mg orally once daily (with a dose escalation by 500-mg increments every 13 weeks, ending with 1000 mg twice daily). The total treatment duration was 52 weeks. Dropout was less than anticipated, with *N =* 56 completing week 52 in the resveratrol arm and *N =* 48 completing week 52 in the placebo arm. Outcomes included safety and tolerability as well as effects on AD biomarkers (plasma Aβ40 and Aβ42, CSF Aβ40, Aβ42, tau, and phospho-tau181) and volumetric MRI (primary outcomes). Clinical outcomes (secondary) were also examined. Detailed pharmacokinetics were obtained in a subset (*n =* 15) at baseline and at weeks 13, 26, 39, and 52. As expected, oral resveratrol was rapidly metabolized with limited bioavailability. However, resveratrol and its major metabolites were measurable in plasma and CSF—demonstrating penetration of the blood-brain barrier. The only significant adverse event was weight loss. Compared to a decline found in the placebo group, plasma Aβ40 and CSF Aβ40 levels were stabilized by resveratrol. In the subset of individuals with biomarker-confirmed AD (baseline Aβ42 <600 ng/ml), resveratrol treatment also stabilized CSF Aβ42. Brain volume loss was increased by resveratrol treatment (3 versus 1%), suggesting a potent anti-inflammatory effect. The activities of daily living scale demonstrated less decline with resveratrol treatment, but the phase 2 study was inadequately powered to determine clinical outcomes. High-dose oral resveratrol is safe and well-tolerated in older individuals with AD. Further studies are needed to interpret the clinical and biomarker changes associated with resveratrol treatment.

### Human Neurodegenerative Disease Magnetic Bead Panels

We used a multiplex Xmap technology that uses magnetic microspheres internally coded with two fluorescent dyes to measure markers of neurodegeneration (Millipore, Cat#: HNABTMAG-68K). All samples including placebo and resveratrol at baseline and 52 weeks were analyzed in parallel using the same reagents. Through precise combinations of these two dyes, multiple proteins are measured within the sample. Each of these spheres is coated with a specific capture antibody. The capture antibody binds to the detection antibody and a reporter molecule, completing the reaction on the surface of the bead. CSF or plasma (25 μl) was incubated overnight at 4 °C with 25 μl of a mixed bead solution, containing human total tau, p-tau181, Aβ42, and Aβ40 (CSF Aβ40 is diluted 1:10). After washing, samples were incubated with 25 μl detection antibody solution for 1.5 h at room temperature. Streptavidin-phycoerythrin (25 μl) was added to each well containing the 25 μl of detection antibody solution. Samples were then washed and suspended in 100 μl of sheath fluid. Samples were then run on MAGPIX with Xponent software. The median fluorescent intensity (MFI) data was analyzed using a 5-parameter logistic or spline curve-fitting method for calculating analyte concentrations in samples. We also performed multiplex ELISA (Millipore, CAT#: HCYTOMAG-60K) to profile a panel of plasma and CSF markers that are indicative of inflammation, including human EGF, FGF-2, Eotaxin, TGF-α, G-CSF, Flt-3L, GM-CSF, Fractalkine, IFNα2, IFNγ, GRO, IL-10, MCP-3, IL-12P40, MDC, IL-12P70, PDGF-AA, IL-13, PDGF-AB/BB, IL-15, sCD40L, IL-17A, IL-1RA, IL-1α, IL-9, IL-1β, IL-2, IL-3, IL-4, IL-5, IL-6, IL-7, IL-8, IP-10, MCP-1, MIP-1α, MIP-1β, RANTES, TNFα, TNFβ, and VEGF.

### Matrix metalloprotease ELISA

Xmap technology uses magnetic microspheres that are internally coded with two fluorescent dyes. Through precise combinations of these two dyes, multiple proteins are simultaneously measured within a sample. Each of these spheres is coated with a specific capture antibody. The capture antibody binds to the detection antibody and a reporter molecule, completing the reaction on the surface of the bead. All samples including placebo and resveratrol at baseline and 52 weeks were analyzed in parallel using the same reagents. A total of 25 μl human CSF or plasma was incubated overnight at 4 °C with 25 μl of a mixed bead solution, containing human matrix metalloproteinase (MMP)-3, MMP-12, and MMP-13 (Millipore Cat# HMMP1MAG-55K) or human MMP-1, MMP-2, MMP-7, MMP-9, and MMP-10 (Millipore Cat# HMMP2MAG-55K). Following extensive washing of the plate, samples were incubated with 25 μl of detection antibody solution for 1.5 h at room temperature and 25 μl of streptavidin-phycoerythrin was added to each well. Samples were then washed and suspended in 100 μl of sheath fluid. Samples were then run on MAGPIX with Xponent software. The median fluorescent intensity (MFI) data was analyzed using a five-parameter logistic or spline curve-fitting method for calculating analyte concentrations in samples according to manufacturer’s protocols.

### Statistical analysis

The inflammatory outcomes measured here are all exploratory, post hoc analyses. Data are summarized as raw values, range as appropriate, and mean ± SD for *N =* 19 in the placebo group and *N =* 19 in the resveratrol group, unless otherwise indicated. All graphs and statistical analyses were performed in Graph Pad Prism Software version 5.01 (Graph Pad Prism Software, Inc. CA. USA). For baseline comparison between the two treatment arms, unpaired *t* tests assuming both equal and unequal variances and Wilcoxon rank sum tests were performed to compare biomarkers and clinical variables. For categorical variables, Pearson’s *χ* ^2^ tests were used for comparison. Paired *t* tests were performed within groups at baseline versus 52 weeks of treatment, and unpaired *t* tests were performed for comparison of placebo and resveratrol treatment. We also fitted simple linear regression to see the associations between cognitive score (MMSE) and each biomarker among all of these individuals. The Benjamini and Hochberg (BH) multiple test correction is applied to control the false discovery rate at 0.05. *p* values (*indicates statistical significance after BH adjustment) are summarized in Tables 1 and 2.

**Table 1:** Summary of statistical tests of null changes between baseline and 52 weeks and tests of null differences at baseline using all detected molecules in CSF of patients treated with placebo (*N =* 19) or resveratrol (*N =* 19)

Analytes	Between baseline and 52 weeks					At baseline		
	Paired *t* test		Unpaired *t* test	Wilcoxon signed rank test		Active vs placebo		
	Placebo	Active		Placebo	Active	Unpaired *t* test (unequal)	Unpaired *t* test (equal)	Wilcoxon signed rank test
Aβ40	0.0031**	0.0014**	0.0784	0.0066**	0.0029**	0.3579	0.3587	0.5284
Aβ42	0.0112*	0.0027**	0.0784	0.0105*	0.0034**	0.3764	0.3739	0.4986
pTau 181	0.0665	0.6498	0.4409	0.0649	0.2891	0.7043	0.7044	0.9319
tTau	0.91	0.3673	0.4614	0.9622	0.2163	0.5769	0.5638	0.9373
MMP-9	0.0474*	0.0027**	0.0241*	0.0771	0.0034**	0.8751	0.8737	1.0000
MMP-3	0.6098	0.5437	0.942	0.5016	0.6848	0.6911	0.6882	0.9311
MMP-2	0.5781	0.2322	0.9759	0.7049	0.3388	0.8828	0.8828	0.8509
MMP-10	0.4965	0.3175	0.9999	0.9341	0.5935	0.9415	0.9410	0.6807
FGF-2	0.3676	0.0248*	0.269	0.2128	0.0273*	0.6751	0.6774	0.5508
TGFα	0.8693	0.0829	0.4257	0.61	0.2324	0.5571	0.5519	0.9843
G-CSF	0.0532	0.1196	0.6722	0.0942	0.1563	0.2042	0.2422	0.4195
MCP-3	0.4751	0.0802	0.2922	0.5625	0.1094	0.2366	0.2305	0.2948
MDC	0.8121	0.001***	0.0505	0.8938	0.0009***	0.0458	0.0566	0.0678
PDGF-AA	0.9866	0.1106	0.0728	0.804	0.0787	0.9883	0.9886	0.8957
IL-4	0.9647	0.0085**	0.1828	0.6875	0.0167*	0.0860	0.0992	0.0748
PDGF-AB/BB	0.1752	0.4337	0.3356	0.0463*	0.057	0.6349	0.6261	0.7881
IL-8	0.262	0.1177	0.1594	0.57	0.0465*	0.9648	0.9648	0.6450
Flt-3L	0.9602	0.1132	0.5639	0.8498	0.155	0.8880	0.8880	0.8109
Fractalkine	0.791	0.4409	0.5973	0.6701	0.9382	0.4541	0.4543	0.8641
IFNα2	0.8832	0.4182	0.2943	0.8361	0.7547	0.8727	0.8729	0.6194
IL-15	0.9469	0.3587	0.3867	0.816	0.4263	0.7049	0.7029	0.6813
IL-7	0.8148	0.4458	0.3147	0.8384	1	0.4650	0.4553	0.4305
IP-10	0.2769	0.4091	0.405	0.6026	0.7368	0.3900	0.3918	0.8509
MCP-1	0.8089	0.5809	0.7419	0.421	0.8971	0.8650	0.8650	0.9864
MIP-1b	0.3973	0.2909	0.6174	0.3621	0.2188	0.7722	0.7632	0.8401

Indicated in bold typeface represents significant associations (at level 0.05) after the Benjamini–Hochberg correction

*p<0.05

**p<0.1

***p<0.001

**Table 2:** Summary of statistical tests of null changes between baseline and 52 weeks and tests of null differences at baseline using all detected molecules in plasma of patients treated with placebo (*N =* 19) or resveratrol (*N =* 19)

Analytes	Between baseline and 52 weeks					At baseline		
	Paired *t* test		Unpaired *t* test	Wilcoxon signed rank test		Baseline: active vs placebo		
	Placebo	Active		Placebo	Active	Unpaired *t* test (unequal)	Unpaired *t* test (equal)	Wilcoxon signed rank test
MMP-9	0.1477	0.8332	0.7577	0.2412	0.6319	0.8751	0.8737	1.0000
MMP-3	0.3193	0.0062**	0.065	0.3575	0.0102*	0.6911	0.6882	0.9311
MMP-2	0.5783	0.1647	0.1671	0.7148	0.177	0.8828	0.8828	0.8509
MMP-10	0.4557	0.0243*	0.0106*	0.5417	0.0353	0.9415	0.9410	0.6807
MMP-12	0.457	0.9138	0.4917	0.4631	0.9306	0.3667	0.3787	0.3310
MMP-13	0.1897	0.3379	0.0489*	0.3575	0.421	0.0598	0.0722	0.0938
MMP-1	0.7483	0.9518	0.7139	0.4263	0.8129	0.1371	0.1264	0.1059
FGF-2	0.6374	0.9743	0.6426	0.7002	0.6701	0.6751	0.6774	0.5508
G-CSF	0.3334	0.4526	0.2682	0.4131	0.5703	0.2042	0.2422	0.4195
MDC	0.7177	0.8208	0.1778	0.7148	0.7605	0.0458	0.0566	0.0678
PDGF-AA	0.8196	0.5719	0.3956	0.583	0.5421	0.9883	0.9886	0.8957
PDGF-AB/BB	0.2176	0.889	0.998	0.3258	0.9246	0.6349	0.6261	0.7881
IL-8	0.2096	0.7207	0.0322*	0.5416	0.9794	0.9648	0.9648	0.6450
EGF	0.5238	0.5667	0.7799	0.4263	0.8564	0.8870	0.8815	0.7234
Eotaxin	0.5987	0.0518	0.0697	0.5016	0.0611	0.7968	0.8017	0.8212
GM-CSF	0.6863	0.4321	0.3562	0.8501	0.4648	0.5001	0.4856	0.6638
Fractalkine	0.8863	0.8397	0.8579	0.9032	0.9245	0.4541	0.4543	0.8641
IFNα2	0.8981	0.8459	0.9661	1	0.9622	0.8727	0.8729	0.6194
IFNy	0.8316	0.438	0.8992	0.7148	0.463	0.8357	0.8406	0.8745
GRO	0.3481	0.6638	0.8992	0.2958	0.9622	0.9496	0.9478	0.5825
IL-12P40	0.6103	0.0485*	0.6473	0.9102	0.0781	0.2033	0.1897	0.3816
IL-12P70	0.5558	0.0404*	0.8159	0.7344	0.0424*	0.6327	0.6141	0.7730
sCD40L	0.3657	0.793	0.9062	0.3258	0.9653	0.6623	0.6483	0.8802
IL-17A	0.3792	0.7593	0.1786	0.4752	0.8158	0.3152	0.2696	0.6923
IL-1RA	0.0462*	0.4993	0.6706	0.0137*	0.7615	0.0880	0.0802	0.0317
IL-2	0.8926	0.0311*	0.9868	1	0.0547	0.2103	0.2015	0.2712
IP-10	0.1619	0.0673	0.0975	0.1353	0.0742	0.3900	0.3918	0.8509
MCP-1	0.5574	0.0637	0.2533	0.7148	0.0523	0.8650	0.8650	0.9864
MIP-1a	0.3696	0.2241	0.588	0.3594	0.1289	0.0175	0.0247	0.0440
MIP-1b	0.3694	0.6581	0.5786	0.3652	0.8469	0.7722	0.7632	0.8401
RANTES	0.8545	0.0335*	0.2919	0.9515	0.0366*	0.9889	0.9889	0.9699
TNFα	0.0221*	0.2091	0.7741	0.0161*	0.463	0.4081	0.4298	0.5861

Indicated in bold typeface represents significant associations (at level 0.05) after the Benjamini–Hochberg correction

*p<0.05

**p<0.1

### Standard protocol approvals, registrations, and patient consents

This study was conducted in accordance with Good Clinical Practice guidelines. Informed consent was obtained from participants and study partners. The study was conducted under local institutional review board supervision, under Food and Drug Administration IND 104205, and registered at ClinicalTrials.gov (NCT01504854).

## Results

### CSF biomarkers

**Fig. 1:**
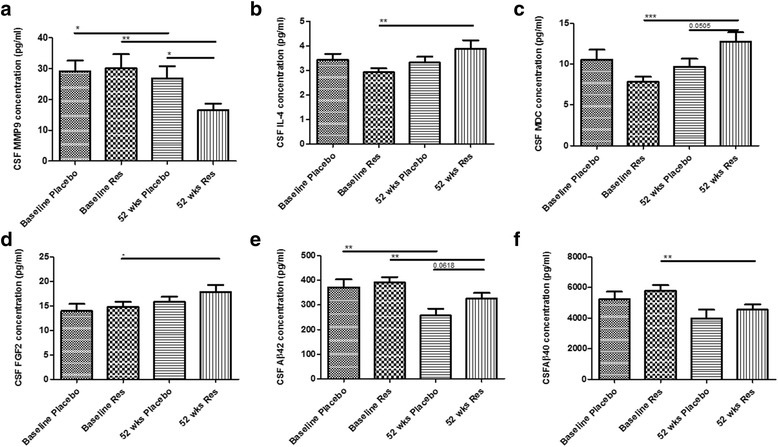
ELISA concentrations of **a** MMP9, **b** IL-4, **c** MDC, **d** FGF2, **e** Aβ42, and **f** Aβ40 in the CSF from patients treated with placebo (*N =* 19) or resveratrol (*N =* 19) for 52 weeks. Mean ± SD, *p* values and statistical methods are listed in Table 1

At baseline, the levels of CSF biomarkers between the placebo group and resveratrol group were not significantly different (Table 1). The level of CSF MMP9 was significantly reduced in the placebo group between baseline and 52 weeks (Fig. 1a), and MMP9 was further reduced (48%) at 52 weeks in the resveratrol group. No change in MMP9 was detected in the plasma (Table 2). Additionally, the level of interleukin (IL)-4 did not change in the placebo group, but CSF IL-4 was increased (Fig. 1b) in the resveratrol group. The CSF levels of macrophage-derived chemokine (MDC) (Fig. 1c) and fibroblast growth factor (FGF)-2 (Fig. 1d) were also increased after 52 weeks of resveratrol treatment compared to baseline, with no changes in these molecules in plasma (Table 1). There was no change in total CSF tau or hyper-phosphorylated (p-tau)181 levels in the resveratrol group and other inflammatory markers (Table 1) did not change. The level of CSF Aβ42 was significantly reduced in the placebo (Fig. 1e) and resveratrol group at 52 weeks compared to baseline, consistent with our previous data [2]. However, the decline of CSF Aβ42 in the placebo group was greater than the decline in the resveratrol group (*p* = 0.0618). Furthermore, CSF Aβ40 was significantly reduced in the resveratrol group at 52 weeks compared to baseline (Fig. 1f). Multiple test corrections to control for a false discovery rate <0.05 were performed and the significant associations in CSF markers were unchanged after this analysis.

### Plasma biomarkers

**Fig. 2:**
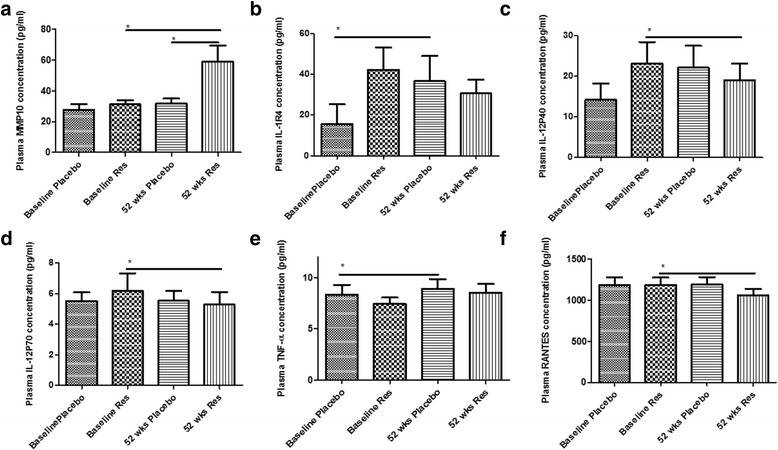
ELISA concentrations of **a** MMP10, **b** IL-1R4, **c** IL-12P40, **d** IL-12P70, **e** TNFα, and **f** RANTES in plasma from patients treated with placebo (*N =* 19) or resveratrol (*N =* 19) for 52 weeks. Mean ± SD, *p* values and statistical methods are listed in Table 2

At baseline plasma level of each biomarker between the placebo group and resveratrol group were not significantly different (Table 2). The plasma level of MMP10 was increased at 52 weeks of resveratrol treatment compared to baseline and placebo (Fig. 2a), and MMP10 did not change in CSF (Table 1). MMP3 and MMP2 did not change in the CSF (Table 1) or plasma and MMP1, MMP12, and MMP13 did not change in plasma (Table 2). Plasma IL-1R4 (Fig. 2b) and IL-12P40 (Fig. 2c) were increased at 52 weeks compared to baseline in the placebo group, but this increase was slightly reduced in the resveratrol group. The plasma levels of IL-12P70 (Fig. 2d) did not change with placebo but was reduced at 52 weeks compared to baseline in the resveratrol group before multiple test adjustment. Plasma tumor necrosis factor (TNF)-α (Fig. 2e) was increased at 52 weeks compared to baseline with placebo and did not change in the resveratrol group. Plasma levels of RANTES/CCL5 (Fig. 2f) did not change with placebo but was reduced at 52 weeks compared to baseline in the resveratrol group. The plasma level of IL-8 (Table 2) was reduced at 52 weeks in the resveratrol group compared to placebo. No changes were observed in other markers (Tables 1 and 2) between groups. However, statistical associations in plasma markers did not hold after multiple test correction, suggesting that samples from a larger number of subjects may be required to discover putative significant effects.

### Cognitive outcomes

**Fig. 3:**
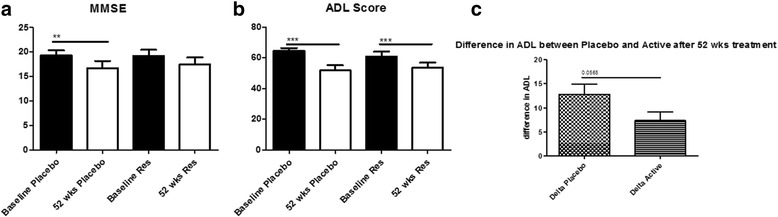
Histograms represent **a** MMSE scores and **b** ADCS-ADL and **c** changes in ADL in placebo versus resveratrol groups in patients treated with placebo (*N =* 19) or resveratrol (*N =* 19) for 52 weeks. Mean ± SD, **p < 0.01, ***p < 0.001

A reduction in mini-mental score examination (MMSE) scores was observed at 52 weeks compared to baseline in the placebo group (Fig. 3a, *p <* 0.01), but no significant change was detected in MMSE between baseline and 52 weeks with resveratrol treatment. ADCS-ADL scores showed a decline at 52 weeks compared to control (Fig. 3b) in both placebo (*p <* 0.001) and resveratrol (*p <* 0.001) groups; however, the decline in placebo was twofold greater than resveratrol at week 52 (Fig. 3c), suggesting that resveratrol may slow progressive cognitive and functional decline in mild to moderate AD subjects. There is no statistically significant association between the change in MMSE and change in each of CSF or plasma biomarker between baseline and 52 weeks (Table 3).

**Table 3:** Summary of statistical tests of associations between changes in MMSE for 52 weeks and changes in each biomarker for 52 among patients treated with placebo (*N =* 19) or resveratrol (*N =* 19)

Association between changes in MMSE and changes in biomarkers			
CSF	*p* value	Plasma	*p* value
Aβ40	0.8367	MMP-9	0.1061
Aβ42	0.2180	MMP-3	0.1358
pTau 181	0.4521	MMP-2	0.4234
tTau	0.6607	MMP-10	0.6379
MMP-9	0.1061	MMP-12	0.9992
MMP-3	0.1358	MMP-13	0.5432
MMP-2	0.4234	MMP-1	0.9490
MMP-10	0.6379	FGF-2	0.5382
FGF-2	0.5382	G-CSF	0.3103
TGFα	0.1270	MDC	0.7157
G-CSF	0.3103	PDGF-AA	0.2266
MCP-3	0.8066	PDGF-AB/BB	0.8598
MDC	0.7157	IL-8	0.0705
PDGF-AA	0.2266	EGF	0.3491
IL-4	0.7163	Eotaxin	0.2782
PDGF-AB/BB	0.8598	GM-CSF	0.9380
IL-8	0.0705	Fractalkine	0.4894
Flt-3L	0.2061	IFNα2	0.9855
Fractalkine	0.4894	IFNy	0.3895
IFNα2	0.9855	GRO	0.2955
IL-15	0.8288	IL-12P40	0.7747
IL-7	0.8204	IL-12P70	0.8371
IP-10	0.2102	sCD40L	0.3075
MCP-1	0.2516	IL-17A	0.8985
MIP-1b	0.5144	IL-1RA	0.5109
		IL-2	0.6367
		IP-10	0.2102
		MCP-1	0.2516
		MIP-1a	0.6162
		MIP-1b	0.5144
		RANTES	0.4541
		TNFα	0.2814

Indicated in bold typeface represents significant associations (at level 0.05) after the Benjamini–Hochberg correction

## Discussion

One of the most striking results of this study is the significant decrease in the level of CSF MMP9 after resveratrol treatment. MMP9 has recently emerged as a major player in several brain pathologies, including neurodegeneration and neuro-inflammation [5]. MMP9 regulates BBB permeability via release of cytokines and free radicals as well as cleavage of vascular basal lamina and/or tight junctions in the neurovascular unit in both MS and AD [6–8]. The decrease in CSF MMP9 levels suggests that resveratrol treatment may reduce CNS permeability and limit the infiltration of leukocytes and other inflammatory agents into the brain. MMP9-mediated breakdown of the basal lamina and destruction of gap junctions in the neurovascular unit result in increased CNS permeability and inflammation in autoimmune encephalitis, hypoxic brain injury, and other diseases [9, 10]. MMP9 knockout reduces neuro-inflammation in experimental autoimmune encephalomyelitis (EAE) [11], while CSF MMP9 is elevated in patients with bacterial meningitis and BBB damage [12]. Moreover, inhibition of MMP9 alleviates the neurological damage associated with human immunodeficiency virus (HIV) infection [13], suggesting that MMP9 activation is a response to HIV infection. These data are also supported by enhanced expression and activity of MMP9 in serum, CSF, and demyelinating lesions in MS [14], and abundant evidence of increased MMP9 expression and activity in ischemic stroke [15, 16]. Animal studies have also revealed significant increases in MMP9 levels after traumatic brain injury [17], but damage to the BBB and behavioral deficits are significantly attenuated in MMP9 knockout animals [18, 19].

MMP9 is highly regulated both spatially and temporally with many target substrates including growth factors, cell surface receptors, and cell adhesion molecules. Low levels of MMP9 messenger RNA (mRNA) and protein expression are detected predominantly in neurons in the hippocampus, cerebellum and cerebral cortex of normal brain [20], but injury significantly increases the mRNA and protein levels and activity of MMP9 [5, 21, 22], which may be derived from brain cells or leukocyte invasion of the brain due to BBB compromise. Intercellular adhesion molecule-5 (ICAM-5), which mediates the regulation of dendritic spine elongation and maturation may be cleaved by MMP9 upon activation of N-methyl-d-aspartate (NMDA) receptors [23, 24], suggesting a role for MMP9 in synaptic function. Furthermore, MMP9 deletion increases the number of CA1 pyramidal neurons and decreases the length and complexity of dendritic spines [25]. Immune system dysfunction may develop with aging in parallel with upregulation of brain MMP9 [26–28]. However, a recent study showed that CSF MMP9 was significantly lower in AD subjects with decreased Aβ42 and Aβ40 and increased total tau and p-tau levels compared to healthy controls [29]. In the current study, the levels of CSF tau and p-tau were not altered by treatment but the levels of CSF Aβ42 and Aβ40 were altered in parallel with a reduction of MMP9. However, there was no difference in the level of CSF MMP9 between placebo and resveratrol-treated groups at baseline, and it is uncertain whether MMP9 in our study population with AD is different from healthy controls. MMP9 activation is likely driven by other MMPs [30], so we examined the level of MMPs in plasma and CSF. Leukocyte penetration into brain parenchyma in EAE models involves β-dystoglycan cleavage that is only abolished in double MMP2 and MMP9 knockout mice [31], suggesting the effects of other MMPs on MMP9 function. MMP10 and MMP3 were slightly increased in the plasma but not CSF of AD patients. MMP9 has overlapping substrates with other MMPs that share similar structures [5], so caution must be used in the interpretation of specific MMP9 targets. MMP9 also plays a role in post-natal brain development during a critical period of synaptic formation and maturation and axonal myelination [32]. In the adult brain MMP9 and MMP3 may be involved in neurogenesis and migratory response mechanisms [33]. MMP9 is upregulated in delayed and acute phases of post ischemic stroke models [34, 35].

MMP9 regulates the CNS immune response due to its ability to activate inflammatory markers and its involvement in BBB maintenance, leading to its regulation of entry of leukocytes into the brain parenchyma [5]. MDC/CCL22 is a small cytokine that belongs to the Cysteine-Cysteine (CC) family and is involved in transport of natural killer cells, chronically activated T lymphocytes (Th2) [36], monocytes, and dendritic cells into injury sites [37]. MDC is expressed in the CNS and is produced by CNS-infiltrating leukocytes and intra-parenchymal microglia in EAE models [38]. Activated microglia secrete MDC that induces chemotaxis of Th2, but not Th1, cells suggesting that MDC produced by microglia regulates neuro-inflammation via recruitment of Th2 cells into the injury site [38]. Leukocyte infiltration into CNS white matter lesions, which contain CD4^+^ and CD8^+^ T cells and activated macrophages/microglia, is a hallmark of MS [39]. Taken together, these findings support the hypothesis that the increase in CNS MDC with resveratrol may facilitate the intracerebral homing of specific leukocytes involved in brain injury in AD, providing a mechanism for responding to amyloid-associated inflammation [40]. MDC is involved in Th2-driven chronic inflammation [41], and this is consistent with the increase of CNS levels of IL-4, which mediates an adaptive immune response via Th2 cell induction [42, 43], leading to a long-term protective immune response. Our results are also consistent with the function MMPs that play an integral role in immune cell development, effector function, migration, and ligand-receptor interactions [44]. T helper cells (Th1 and Th2) secrete MMP9 [45], which plays a critical role in the migration of T cells from the blood stream to the brain and other tissues [46, 47]. Additionally, recent advances in neuro-inflammation implicate abnormal neurotrophic factor signaling, including fibroblast growth factors (FGFs) in HIV-associated neurocognitive decline (HAND) [48] and stroke [49]. The increase in CNS FGF levels after resveratrol treatment suggests an effect on growth factors, which may play a role in neuro-resilience in aging and AD.

Neuro-inflammation may contribute to cognitive impairment and play a significant role in AD progression. Activation of specific microglia/macrophage may be neuroprotective. Although resveratrol treatment did not affect CSF tau, resveratrol significantly attenuated the declines in CSF Aβ42 and Aβ40 levels (compared to placebo) and attenuated cognitive and functional decline (MMSE and ADCS-ADL) between the placebo and treated groups. Resveratrol also reduced the plasma levels of pro-inflammatory makers including IL-1R4, IL-12P40, IL-12P70, TNF-α, and RANTES, independent of CSF changes of the levels of these biomarkers.

Innate immune cells, including CNS resident microglia and peripheral bone marrow-derived macrophages can exhibit a dysfunctional or senescent profile characterized by impaired phagocytosis as AD progresses, indicating that modification of the microglia/macrophage activation state, instead of inhibiting their function, may hold therapeutic promise in AD [50–52]. Resveratrol may facilitate activation of microglia/macrophages therefore inducing a long-term adaptive immune response that may be clinically beneficial in AD subjects. Major impediments of current immunotherapy approaches to AD include limited evidence of significant clinical benefits, and the risk of excessive neuro-inflammation [53].

## Conclusions

Resveratrol may maintain the integrity of the BBB via reduction of MMP9 and induce adaptive immune responses that may promote brain resilience to amyloid deposition. Resveratrol may slow cognitive decline in AD via a coordinated peripheral and central immune response that may also arrest neuronal death. In conclusion, the exploratory findings of the current study encourage further validation of the hypothesis that resveratrol may seal off a leaky BBB and contribute to cognitive and functional improvement in a larger follow-up study with AD patients.

## Abbreviations

AD: Alzheimer’s disease
BBB: Blood-brain barrier
CR: Caloric restriction
CSF: Cerebrospinal fluid
IL: Interleukin
MDC: Macrophage-derived chemokine
MMP: Matrix metalloproteinase
Res: Resveratrol
SIRT: Surtuin
TNF-α: Tumor necrosis factor-α
